# Two Complete Genome Sequences of Phasey Bean Mild Yellows Virus, a Novel Member of the *Luteoviridae* from Australia

**DOI:** 10.1128/genomeA.01569-15

**Published:** 2016-02-04

**Authors:** Murray Sharman, Monica Kehoe, Brenda Coutts, Joop van Leur, Fiona Filardo, John Thomas

**Affiliations:** a Department of Agriculture and Fisheries, Brisbane, Queensland, Australia; b Department of Agriculture and Food, Perth, Western Australia, Australia; c Department of Primary Industries, Tamworth, New South Wales, Australia; d The University of Queensland, Brisbane, Queensland, Australia

## Abstract

We present here the complete genome sequences of a novel polerovirus from *Trifolium subterraneum* (subterranean clover) and *Cicer arietinum* (chickpea) and compare these to a partial viral genome sequence obtained from *Macroptilium lathyroides* (phasey bean). We propose the name phasey bean mild yellows virus for this novel polerovirus.

## GENOME ANNOUNCEMENT

The family *Luteoviridae* consists of three separate genera, *Luteovirus*, *Enamovirus*, and *Polerovirus*. Each contains either 5 or 6 open reading frames (ORFs), but only four of these are common to all genera (1–3). One of the genomic features that distinguishes poleroviruses is the presence of both ORF0 and ORF4.

One sample of *Trifolium subterraneum* (subterranean clover) collected in 2013 from Esperance, Western Australia (isolate ESPCL15), and one sample of *Cicer arietinum* (chickpea) collected in 2012 from Liverpool Plains, New South Wales, Australia (isolate NSWCP15), were extracted for total RNA using the Spectrum plant total RNA kit (Sigma-Aldrich, Australia). The total RNA extracts were sent to the Australian Genome Research Facility (AGRF) for library preparation and 100-bp paired-end sequencing on an Illumina HiSeq 2000. The reads from each sample were assembled and the genomes annotated using CLC Genomics Workbench 6.5 (CLC bio) and Geneious 6.1.6 (Biomatters), as described by Kehoe et al. (4).

Samples of *Macroptilium lathyroides* (phasey bean) with mild yellowing symptoms were collected in 2011 from Emerald, Queensland, Australia. A reference sample (isolate 2805) was lodged in the Queensland Government plant virus collection. From this, total RNA was extracted using a BioSprint 15 plant DNA kit (Qiagen), as per the manufacturer’s instructions, except that RNase A was not used. PCR products were amplified using previously described conditions and cycling parameters (5), using the overlapping primer pairs PLF/PLR, Pol3167F/Pol3982R, and Pol3870F/AS3 (5–7), and were sequenced directly using an Applied Biosystems automated sequencing system (AGRF, Brisbane, Australia). After the removal of the primer sequences, the resulting partial genome fragment from isolate 2805 was 1,360 nucleotides (nt) in length.

The two complete genomic sequences shared a nucleotide identity of 91.9%. The 1,360-nt partial sequence for isolate 2805 had 96.4% nucleotide identity to isolate ESPCL15 and 95.3% nucleotide identity to isolate NSWCP15. Over a 585-bp region, the closest match by BLAST (8) in GenBank for isolate 2805 was 97% nucleotide identity to HQ543091, which was referred to as a cucurbit aphid-borne yellows virus (CABYV)-like isolate from Tasmania, Australia (9). However, HQ543091 is distinct from CABYV, with only 81% nucleotide identity to the nearest confirmed isolate, EF029113 (10). Furthermore, the closest matches by BLAST for isolates ESPCL15 and NSWCP15 were only 71 to 72% nucleotide identity to CABYV accession numbers X76931 and LC082306, respectively, over an approximately 3,000-bp region. Both genomes have the combination of ORF0 and ORF4, which is typical of poleroviruses. The two complete genome sequences from isolates ESPCL15 and NSWCP15, the partial sequence from isolate 2805, and the CABYV-like isolate from Tasmania (HQ543091) appear to all be members of the same species, a novel polerovirus, which we propose to name phasey bean mild yellows virus.

### Nucleotide sequence accession numbers.

The sequences were deposited in GenBank with the accession numbers KT962999 (isolate NSWCP15), KT963000 (isolate ESPCL15), and KT906372 (isolate 2805).
